# Disaggregation of Dairy in Composite Foods in the United Kingdom

**DOI:** 10.1016/j.cdnut.2024.103774

**Published:** 2024-05-16

**Authors:** Lindsay M Jaacks, Birdem Amoutzopoulos, Ricki Runions, Alexander Vonderschmidt, Geraldine McNeill, Fiona Comrie, Alana McDonald, Polly Page, Cristina Stewart

**Affiliations:** 1 Global Academy of Agriculture and Food Systems, University of Edinburgh, Midlothian, United Kingdom; 2 MRC Epidemiology Unit, University of Cambridge, Cambridge, United Kingdom; 3 Food Standards Scotland, Aberdeen, United Kingdom; 4 MRC/CSO Social and Public Health Sciences Unit, School of Health and Wellbeing, University of Glasgow, United Kingdom

**Keywords:** dairy products, composite foods, food groups, food composition, dietary assessment, 24-hour dietary recall, measurement error, diet monitoring, sustainable diets, Europe

## Abstract

Dairy, especially cheese, is associated with high levels of greenhouse gas emissions. Accurate estimates of dairy consumption are therefore important for monitoring dietary transition targets. Previous studies found that disaggregating the meat out of composite foods significantly impacts estimates of meat consumption. Our objective was to determine whether disaggregating the dairy out of composite foods impacts estimates of dairy consumption in Scotland. Approximately 32% of foods in the UK Nutrient Databank contain some dairy. In the 2021 Scottish Health Survey, mean daily intakes of dairy with and without disaggregation of composite foods were 238.6 and 218.4 g, respectively. This translates into an 8% underestimation of dairy consumption when not accounting for dairy in composite foods. In particular, milk was underestimated by 7% and cheese and butter by 50%, whereas yogurt was overestimated by 15% and cream by 79%. Failing to disaggregate dairy from composite foods may underestimate dairy consumption.

## Introduction

Food systems account for nearly one-third of greenhouse gas emissions globally [1], and thus, transitioning to more sustainable diets plays an important role in climate change mitigation. Dairy, especially cheese, is associated with high emissions relative to other animal-source foods such as fish, poultry, and eggs [2]. However, prospective cohort studies suggest dairy is moderately protective against type 2 diabetes and colorectal cancer, although the evidence is inconsistent [[3], [4], [5]]. A systematic review of randomized controlled trials found that 4 of the 10 studies reported dairy consumption improved insulin sensitivity, 1 of the 10 reported a negative effect, and 5 of the 10 reported no effect [6]. These findings were supported by a more recent network meta-analysis that concluded there was low certainty of an effect of dairy on glycemia [7]. In terms of nutrition, dairy is an important source of essential vitamins such as vitamins B-12, riboflavin, A, and D and minerals such as calcium, iodine, phosphorus, and potassium [[8], [9], [10], [11]].

The accurate estimation of dairy is important for monitoring dietary transition targets. Previous studies have found that disaggregating the meat out of composite foods can have a significant impact on estimates of meat intake. For example, studies in the United Kingdom, Ireland, and United States have shown that failing to disaggregate meat from composite foods substantially overestimates meat intake [[12], [13], [14], [15]]. Dairy is also often consumed as part of composite foods such as cheese in a lasagna or milk in pancakes. Moreover, dairy foods such as yogurt or ice cream that are classified as 100% dairy may have some proportion of their ingredients as nondairy items such as fruit, nuts, and sugar. Thus, failing to disaggregate dairy from composite foods may also substantially influence estimates of dairy intake.

Our objective was to disaggregate dairy out of composite foods in order to improve the estimation of dairy consumption and the contribution of dairy to nutrient intake in Scotland. This is especially timely in light of the fact that the Scottish Government has recently partially accepted the Climate Change Committee’s recommendation to reduce meat and dairy by 20% by 2030 [16].

## Methods

Our study estimated dairy consumption among adults aged ≥16 y in the nationally representative Scottish Health Survey (SHeS) in 2021 [17]. Ethical approval for SHeS 2021 was obtained from the Health and Care Research Ethics Committee for Wales (reference number: 17/WA/0371). SHeS is designed to provide data at national level about the population living in private households in Scotland. Royal Mail’s small user Postcode Address File was used as the sample frame. The required number of addresses for each stratum (local authorities) were sampled from the sample frame using systematic random sampling with addresses within primary sampling units (data zones on the islands and intermediate geographies elsewhere) ordered by urban-rural classification, Scottish Index of Multiple Deprivation rank, and postcode. All adults aged ≥16 y in selected households were eligible for interview. The survey weights account for the probability of selection and nonresponse to the survey.

Dietary intake was assessed in adults aged ≥16 y using up to two 24-h dietary recalls self-administered via Intake24 (https://intake24.org/) [17]. Intake24 derives the nutrient content of foods reported using the UK Nutrient Databank (NDB) [18]. We anticipated using the disaggregated dairy values for future studies collecting dietary recalls using the NDB integrated into Intake24. Thus, we created a food list for disaggregation that included all unique foods reported in SHeS 2021 (*n* = 1884) as well as unique foods not reported as consumed in SHeS 2021 but in the NDB and thus may be reported in future surveys using Intake24 (*n* = 543). The total number of unique foods for disaggregation was 2427.

We merged these unique foods with the Food Standards Agency Standard Recipes Database (SRD), a hierarchical database that includes ingredient-level information for both homemade and manufactured foods [19]. Only 6 unique foods that were likely to contain dairy did not have a match in the SRD and were assigned a nearest neighbor match as a substitute (Supplemental Table 1). In 2 instances, there were matches with higher specificity in the SRD than in the NDB, and the dairy content was taken from the more specific SRD code (Supplemental Table 2). In 1 instance, there was higher specificity in the NDB than that in the SRD, and the dairy content was taken from the more specific NDB (Supplemental Table 2). In several instances, modifications were made to food codes in order to improve the accuracy of matches between the NDB and SHeS and the SRD (Supplemental Table 3).

There were 1097 unique ingredients in the 2427 foods. Two authors (LMJ and AV) independently reviewed and classified the list of 1097 ingredients as dairy compared with nondairy. Any discrepancies in classification were discussed with a third author (GM) until consensus was reached. A total of 89 of these ingredients were classified as dairy. These dairy ingredients were further classified as milk, cheese, yogurt, cream, or butter (Supplemental Tables 4–6). Milk, cheese, and yogurt were further classified as skimmed, semiskimmed, or full fat. Cream was further classified as semiskimmed or full fat. Cheese was further classified as cheddar, cottage cheese, or other. Toddler milks (e.g., SMA, Aptamil, and Cow and Gate) were classified as full fat milk. Protein powder, Ensure liquid, and cream liqueurs were not classified as dairy. Thus, estimates are for dairy from foods and beverages not including supplements.

The amount of dairy ingredients in 100 g of the recipe was summed within a recipe to derive the total amount (in grams) of dairy in that recipe per 100 g. This was repeated for each dairy subtype.

Dairy disaggregation was conducted using R version 4.2.2 (2022-10-31). SHeS analyses to derive mean daily consumption (in grams) of dairy and dairy subtypes, per capita (i.e., including both consumers and nonconsumers of dairy), without and with disaggregation, were conducted using Stata IC Version 17 and were weighted to be representative of adults aged ≥16 y living in Scotland. The age range for SHeS 2021 was 16–95 y.

## Results

**FIGURE 1:**
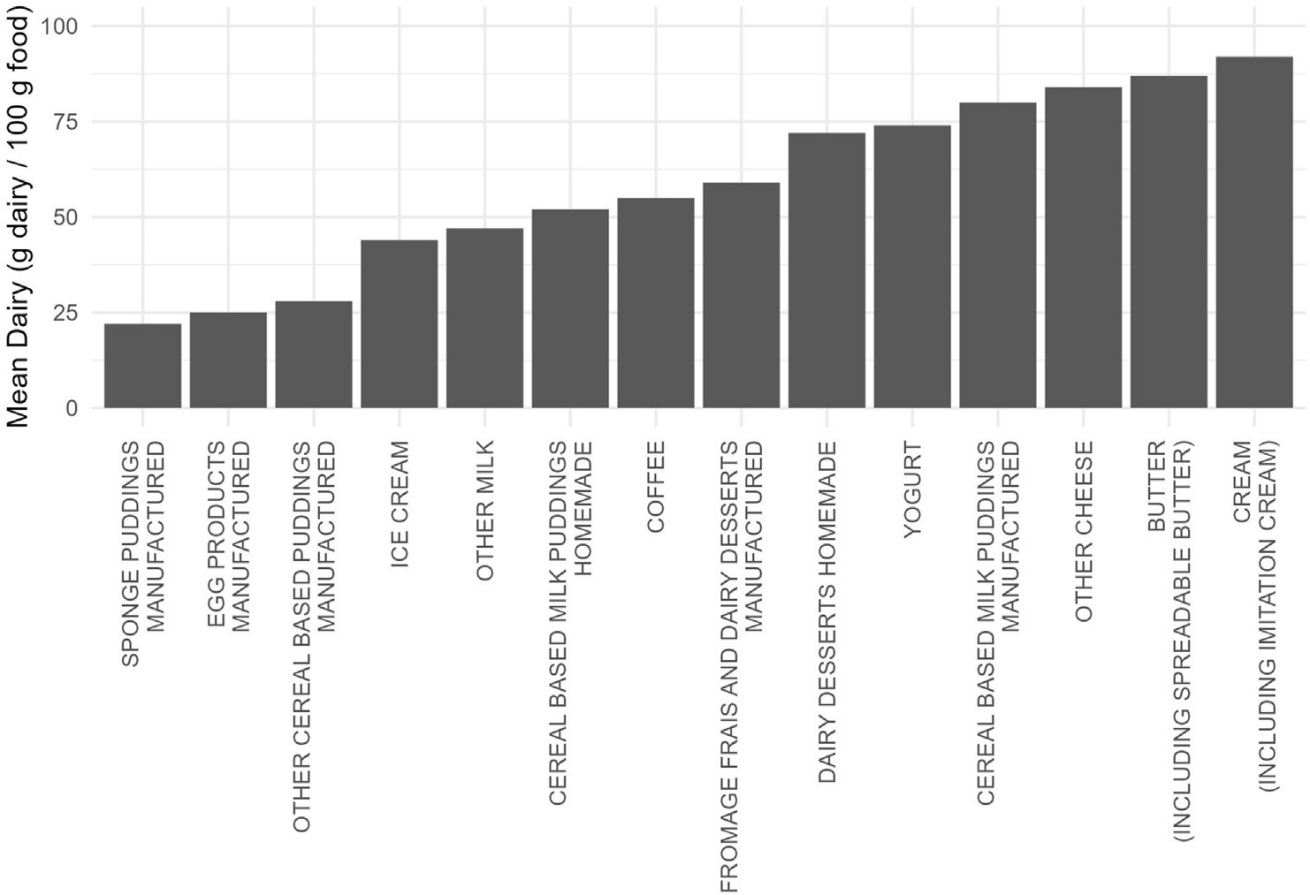
Mean dairy (in grams) per 100 g food for subfood groups with >20% and <100% dairy. Butter includes spreadable butter, which contains 63 g butter per 100-g product.

A total of 69 of the 2427 foods (3%) were 100% dairy items. A total of 1653 (68%) did not contain any dairy. The remaining 705 (29%) were dairy-containing composite foods. The amount of dairy in composite foods ranged from 0.01 g/100 g of “chicken curry, ready meal, with rice, reduced fat” to 98.44 g/100 g of “butter, salted.” Besides dairy foods (e.g., milk, yogurt, cheese, cream, and butter), dairy was most commonly found in cereal-based milk puddings, dairy desserts, coffees, and manufactured egg products including ready meals (e.g., quiches and Pavlova) (Figure 1).

**FIGURE 2:**
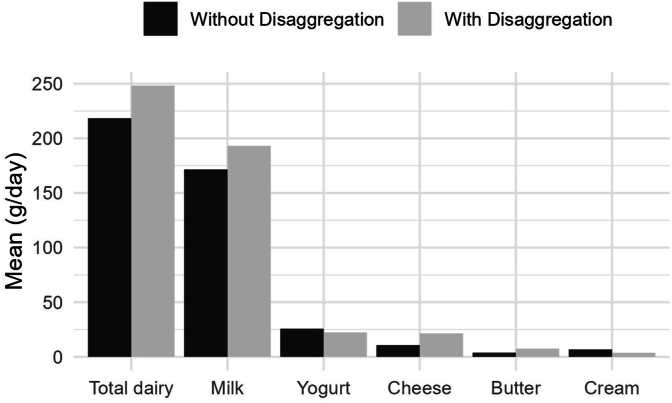
Mean intakes (in grams) per day of dairy and dairy subtypes, per capita, among adults aged 16 y and older participating in the Scottish Health Survey (2021).

In SHeS 2021, the mean (SD) per capita intakes of dairy with and without disaggregation of composite foods were 238.6 g (207.5 g) and 218.4 g (218.0 g) per day, respectively. This translates into an 8% underestimation of mean dairy intake when disaggregation of composite foods is not taken into account. In particular, milk was underestimated by 7%, and cheese and butter by 50%, whereas yogurt was overestimated by 15% and cream by 79% (Figure 2). The large overestimation of cream was due to ice cream and other dairy desserts (e.g., ice cream including a wafer cone) being included in estimates of cream without disaggregation. In the disaggregated data, the cream content of ice cream ranged from 7 g/100 g product for “Magnum classic or white” to 40 g/100 g product for “Kulfi, Indian ice cream.”

Nearly three-quarters (74%) of total dairy intake came from noncomposite dairy products, including milk (44% of total dairy intake), cheese, yogurt, cream, butter, ice cream, and dairy desserts (Supplemental Table 7). The remaining 26% of total dairy intake came from composite foods, especially pasta, rice, and other cereals (5.4%) and sandwiches (3.5%). Sweets (biscuits, buns, cakes, pastries, fruit pies, puddings, sugars, preservers, sweet spreads, chocolate, confectionery, and ice cream) accounted for 6.7% of dairy.

## Discussion

Dairy consumption is likely to be underestimated if dairy from composite foods is not captured. In our analysis of a representative sample of adults living in Scotland, this underestimation was, on average, 8% by weight. However, there was substantial variability in estimation across dairy subtypes with cheese and butter being underestimated by 50%. Given that cheese has one of the highest greenhouse gas emissions intensities of any food product—only beef, lamb, and farmed shrimp are higher in terms of average emissions intensities per 100 g protein [2]—this has potentially important implications for estimating the emissions attributable to diets. Assuming a carbon footprint of 1.2 kg CO_2_e/L of milk produced in the United Kingdom [20] and milk utilization by dairies data from the UK Government [21], the underestimation of dairy consumption if dairy from composite foods is not captured would translate into a 37% underestimation of the greenhouse gas emissions associated with dairy consumed by adults living in Scotland (an estimated 184 kg CO_2_e/kg per capita per year with disaggregation compared with 134 kg CO_2_e/kg per capita per year without disaggregation).

Our findings for dairy contrast to findings for meat. Previous studies in the United Kingdom, Ireland, and United States found that failing to disaggregate meat from composite foods substantially overestimates meat intake, as composite foods containing meat were reported as 100% meat before disaggregation [[12], [13], [14], [15]]. For example, in an average portion of Bolognese sauce (145 g), there is an estimated 81 g meat, so failing to disaggregate would result in an overestimation of meat by 44% by weight [13]. In the same way, some dairy foods are assumed to be 100% dairy when in fact they are not—for example, fruit flavored yogurts and ice cream, leading to an overestimation of dairy from these foods. In contrast, some composite foods contain dairy that would otherwise not be captured in an analysis of “dairy foods,” e.g., cheese on pizza. As such, the degree of underestimation (or overestimation of dairy will depend on the consumption patterns of these dairy-containing foods in the population of interest.

To the best of our knowledge, this is one of the first studies to estimate population-level dairy consumption without and with disaggregation of dairy from composite foods. One previous analysis looked at trends in low-fat and high-fat dairy intake in the UK National Diet and Nutrition Survey from 2008/2009 to 2015/2016, accounting for dairy in composite foods using an older version of the Food Standards Agency SRD [22]. Similar to our study, that thesis found that milk was the largest contributor to dairy [22]. However, they did not report on what proportion of dairy came from dairy foods versus composite foods [22]. Although the USDA’s Food Patterns Equivalents Database converts the foods and beverages reported in the NHANES into cup equivalents of dairy, it is only for milk, cheese, and yogurt [23]. Butter and cream are included in an aggregate category for solid fats that includes other nondairy fats, as well as the fat naturally present in milk, yogurt, and cheese in excess of 1.5 g per cup equivalent. Moreover, milk includes calcium-fortified soy milk. Thus, our results cannot be directly compared with those of NHANES. Nonetheless, it is worth pointing out that a study by the National Dairy Council found that 48% of dairy consumed by adults in NHANES was from “other foods” such as composite foods (e.g., pizza) and foods containing dairy (e.g., desserts) [8]. This is substantially higher than estimated for Scotland in our study (26%) and highlights the need for population-specific studies on patterns of food consumption.

Dairy is a common ingredient in the United Kingdom. One-third of foods in the NBD, which underlies diet monitoring (e.g., the National Diet and Nutrition Survey) and many widely used nutritional epidemiological studies (e.g., the UK Biobank) across the United Kingdom, contain some dairy. Although this work was initially completed to estimate dairy consumption among adults in Scotland, the resulting database, publicly available on GitHub, can be used for any data sets linked to the NDB.

There are several limitations of our approach worth noting. The specificity of the disaggregated dairy is limited by the specificity of reported dietary intake data and the specificity of NDB. For example, all plain croissants are assumed to have 25 g butter per 100-g croissant. If instead a respondent had a croissant made with a vegetable fat spread (i.e., shortening), we would overestimate their dairy intake. As the availability and use of vegan dairy substitutes increases, the specificity of dietary intake data and corresponding nutrient databases will need to increase to accommodate these shifts. We used the Food Standards Agency SRD for data on grams of ingredients [19]. However, there is likely to be variability in the grams of dairy ingredients in a given food, for both homemade and manufactured foods. For example, 19 participants (0.6% of the sample) in SHeS 2021 reported consuming homemade Yorkshire pudding. The Food Standards Agency SRD includes about 60-g semiskimmed milk in 100-g homemade Yorkshire pudding. However, a meta-analysis of 33 Yorkshire pudding recipes suggests that there can be considerable variability in the amount of milk in a portion of Yorkshire pudding [24]. Capturing this variability would require greater specificity in reported dietary intake data including the recipe for all homemade dishes and the ingredients panel for all manufactured dishes. Such data are not available for most national dietary surveys. Future work could consider exploring disaggregation of dairy from mixed dishes using alternative open-source recipe databases such as RecipeDB, which pooled 118,171 recipes from http://www.food.com and http://allrecipes.com [25]. The disaggregated dairy in our database is based on the estimated amount of dairy ingredients before cooking for homemade foods. For example, it includes the amount of cream in spaghetti carbonara as listed in ingredients for a recipe for spaghetti carbonara. It is not the amount of cream in the final product as consumed. We felt that this was more appropriate because the environmental impacts arise from the product as produced, rather than as consumed. However, the nutritional value to the individual arises from the product as consumed, so estimating the nutritional contribution of dairy should take water loss into account, especially for milk in composite foods. Finally, to translate our findings on dairy consumption into dairy production would require further converting the yogurt, cheese, cream, and butter into milk equivalents. Milk utilization by dairies is available for England and Wales from the Department for Environment, Food, and Rural Affairs [21]. For example, the production of 1 kg of cheddar requires 9.5 L of milk [21]. Thus, such conversions are possible for those interested in understanding how the consumption patterns described in this study may impact food production systems.

Approximately 26% of dairy consumed in Scotland comes from composite foods such as pasta mixed dishes, pizza, sandwiches, biscuits, and chocolate. Although this means that failing to account for dairy in these foods will underestimate dairy consumption, it also means that the majority of dairy comes from noncomposite foods such as milk. As such, only addressing foods high in fat, salt, and sugar—which are the focus of many food policies in the UK [26]—such as chocolate, biscuits, and pizza, will unfortunately not be sufficient to achieve sustainable dietary transition targets such as the one set by the Climate Change Committee to reduce dairy by 20% by 2030 [16]. Further work is needed to understand optimal dietary transitions for dairy.

## Author contributions

The authors responsibilities were as follows – LMJ, BA, RR, CS, AV, GM: contributed to the research design; LMJ, CS: conducted the statistical analyses with feedback from BA; LMJ, RR, CS, AV, GM: directly accessed and verified the underlying data; LMJ: wrote the first draft of the manuscript; and all authors: critically reviewed the manuscript and approved the final version.

## Conflicts of interest

The authors report no conflicts of interest.

## Funding

This research was funded by Food Standards Scotland (award number FSS/2021/012). Food Standards Scotland were involved in the study design, writing of the manuscript, and the decision to submit the manuscript for publication. LMJ is funded by Medical Research Council/UK Research and Innovation (Grant Ref: MR/T044527/1). For the purpose of open access, the author has applied a Creative Commons Attribution (CC BY) license to any Author Accepted Manuscript version arising from this submission.

## Data sharing

Data from the Scottish Health Survey can be accessed on the UK Data Service (https://ukdataservice.ac.uk/). All analysis codes and files are publicly and freely available on GitHub: https://github.com/Cristina-Stewart/SHeS_Dairy-disaggregation.
