# Supplementary material for: Disaggregation of Dairy in Composite Foods in the United Kingdom
Source: Curr Dev Nutr. 2024 May 16;8(8):103774. doi: 10.1016/j.cdnut.2024.103774 (PMC11325663; doi:10.1016/j.cdnut.2024.103774)
Supplement: Multimedia component 1 [file mmc1.docx]

**Disaggregation of Dairy in Composite Foods in the UK**

Lindsay M Jaacks^1^, Birdem Amoutzopoulos^2^, Ricki Runions^1^, Alexander Vonderschmidt^1^, Geraldine McNeill^1^, Fiona Comrie^3^, Alana McDonald^3^, Polly Page^2^, Cristina Stewart^1^

^1^Global Academy of Agriculture and Food Systems, University of Edinburgh, Midlothian, UK

^2^MRC Epidemiology Unit, University of Cambridge, Cambridge, UK

^3^Food Standards Scotland, Aberdeen, UK

| **Supplemental Table S1.** Foods missing in the Food Standards Agency (FSA) Recipes Database and nearest-neighbor substitution used for disaggregation of dairy. Items shaded in grey were assumed to not contain dairy and no substitute was assigned. | | | |
| --- | --- | --- | --- |
| **Food Number** | **Food Description** | **Nearest-Neighbor Substitution** | |
|  |  | **Food Number** | **Food Description** |
| 11395 | Aubergine puree |  |  |
| 11396 | Cucumber puree |  |  |
| 11398 | Rhubarb, pureed |  |  |
| 11408 | Grapefruit puree |  |  |
| 11413 | Wellkid multivitamin liquid (age 4-12) |  |  |
| 11585 | Children’s' multivitamin and minerals |  |  |
| 11586 | Children’s' chewable multivitamins |  |  |
| 11416 | Chocolate covered fingers (e.g., Cadbury milk chocolate fingers) | 2276 | Twix (chocolate & caramel biscuit fingers) |
| 11422 | Toblerone | 2254 | Milk chocolate bar (e.g., Dairy Milk) |
| 11419 | Oreos, including supermarket brands | 268 | Biscuits filled with cream (e.g., bourbons, Fox's crunch creams) |
| 11417 | Mcvities digestive | 259 | Digestive biscuit |
| 11418 | Mcvities chocolate digestive | 260 | Chocolate digestive, biscuit |
| 11420 | Mcvities rich tea | 269 | Semi-sweet biscuit (e.g., Rich Tea, Morning Coffee) |

| **Supplemental Table S2.** Summary of where dairy estimates were taken from in cases where either the UK Nutrient Databank (NDB) or Standard Recipes Database (SRD) had greater specificity than the other. | |
| --- | --- |
| Greater specificity in SRD than NDB | - Vegetable soup, celery soup, parsnip soup, pumpkin soup and butternut squash soup all have the same food code (5607) in the NDB, but separate food codes are available in the SRD for vegetable soup without potato (5607) and butternut squash soup (3955, which contains dairy) - Swede, carrot and swede mash and swede mashed all have the same food code (1921) in the NDB, but separate food codes are available in the SRD for carrot and swede mash (11602, which contains dairy) and boiled swede (1921) |
| Greater specificity in NDB than SRD | - Horlicks/Ovaltine, made with alternative milks (e.g., soya milk) has the food code 10724 and is therefore linked to hot chocolate made with semi-skimmed milk in the SRD. |

| **Supplemental Table S3.** Modifications to food number to improve the accuracy of matches between the UK Nutrient Databank (NDB), Scottish Health Survey (SHeS, 2021) and Food Standards Agency Standard Recipes Database (SRD) for dairy disaggregation. | | | | | |
| --- | --- | --- | --- | --- | --- |
| **Food Number from NDB** | **Food Description**  **Intake24** | **Food Description SHeS 2021** | **Food Description SRD** | **Remarks** | **Edits in R** |
| 120 | Kingsmill sandwich thins, white | Kingsmill sandwich thins, white | BREAD, WHITE SLICED, NOT FORTIFIED | NDB has different food codes / nutrient values for white and wholemeal sandwich thins but SRD only has recipe for wholemeal sandwich thins. | None |
| 11784 | Sandwich thins, wholemeal (brown) |  |  |  | Assigned 'Sandwich thins, wholemeal (brown)' FoodNumber to 11282 in NDB |
| 11282 |  | Sandwich thins, wholemeal (brown) | BREAD SANDWICH WHOLEMEAL THINS KINGSMILL |  | None |
| 262 | Florentines | Florentines | FRUIT BISCUITS NOT WHOLEMEAL | SRD has FLORENTINES, L0FoodCode==330. | Changed FoodNumber to 330 in SHeS and NDB |
| 303 | Chelsea bun | Belgian bun | CHELSEA BUNS NOT WHOLEMEAL | Belgian buns are iced. Belgian bun in SHeS should be linked to Iced Bun in SRD. | Assigned 'Belgian bun' FoodNumber 8125 in SHeS |
| 8125 | Belgian bun | Iced bun | ICED BUN PURCHASED |  | None |
| 574 | Chocolate trifle | Chocolate trifle | TRIFLE, FRUIT PURCHASED WITH FRESH CREAM | All trifle has same food code in NDB. SRD has TRIFLE, CHOCOLATE, PURCHASED, L0FoodCode==6964. | Changed FoodNumber to 6964 in SHeS and NDB |
| 586 | Fruit fritters | Fruit pie, fried (e.g., McDonalds apple pie) | FRUIT PIE/FRIED EG. MC DONALDS | Fruit fritters and Fruit pie, fried have the same food code in NDB. SRD has FRUIT FRITTER FRIED BLEN OIL, L0FoodCode==577. | Assigned 'Fruit fritters' FoodNumber 577 in NDB |
| 701 | Coconut-based yoghurt, fruit, e.g., The Coconut Collaborative | Fruit yoghurt | YOGURT, WHOLE MILK, FRUIT | There is no coconut-based yogurt in the SRD and so no change was made. | None |
| 702 | Coconut-based yoghurt, natural, e.g., The Coconut Collaborative, Koko | Kefir | YOGURT, WHOLE MILK, NATURAL, UNSWEETENED |  | None |
| 7741 | Coconut-based yoghurt, Greek style, e.g., Oykos dairy free, Alpro | Natural yoghurt, Greek-style | YOGURT, GREEK STYLE, COWS, NATURAL, WHOLE MILK |  | None |
| 1921 | Swede, cooked | Carrot and swede mash | SWEDE BOILED | NDB and SRD use 11602 for ‘Carrot and swede mash’. | Assigned 'Carrot and swede mash' FoodNumber 11602 in SHeS |
| 11602 | Carrot and swede mash |  | CARROT AND SWEDE MASH |  | None |
| 2214 | Agave syrup/nectar | Agave syrup/nectar | HONEY (IN JARS) | Honey and agave have same food code in NDB and so no change was made. | None |
| 2257 | Chocolate covered ginger | Chocolate covered ginger | DARK CHOCOLATE WITH CREME OR MINT FONDANT CENTRE EG.AFTER EIGHTS | SRD does not have chocolate covered ginger. Closest match is CRYSTALLISED GINGER, L0FoodCode==10595 which does not contain dairy and so no change was made. | None |
| 2445 | Fish sauce | Fish sauce | SOY SAUCE LIGHT | No fish sauce in SRD. Closest match in SRD is OYSTER SAUCE, L0FoodCode==2438. | Changed FoodNumber to 2438 in SHeS and NDB |
| 2700 | Natural fromage frais, fat free, sugar free | Natural yoghurt, fat free/very low fat (e.g., Total 0% fat Greek yoghurt) | YOGURT, VIRTUALLY FAT FREE, NATURAL, UNSWEETENED | SRD has FROMAGE FRAIS VIRTUALLY FAT FREE, FRUIT, NO ARTFICIAL SWEETNER, L0FoodCode==7739 and FROMAGE FRAIS, LOW FAT, FRUIT, UNFORTIFIED, L0FoodCode==5254 though both contain fruit. The SRD also has 'FROMAGE FRAIS BASED DIP, LOW FAT, L0FoodCode==3781 and PETIT FILOUS FROMAGE FRAIS, L0FoodCode==10218.  SHeS has 'Fromage frais, fruit (incl. children's) not fortified (e.g., Tesco value) 5254' which may be a better fit, but does include fruit.  As there was no clear better match, no change was made | None |
| 2711 | Kumquats | Physalis (Cape Gooseberry) | PHYSALIS (CAPE GOOSEBERRY) | SHeS has 'Orange 2092' and 'Tangerines / mandarins / clementines/ satsumas 2157'. SRD also uses 2157 for Tangerines / mandarins / clementines/ satsumas. | Assigned 'Kumquats' FoodNumber 2157 in NDB |
| 3797 | Instant porridge pot, plain (e.g., Oat So Simple original), made up with water | Instant porridge pot, plain (e.g., Oat So Simple original), made up with water | PORRIDGE MADE SEMISKIMMED MILK | SRD has PORRIDGE MADE UP WITH WATER NO SALT ADDED, L0FoodCode==10284 and PORRIDGE MADE WITH WATER AND ADDED SALT, L0FoodCode==215. | Changed FoodNumber to 10284 in SHeS and NDB |
| 5201 | Cake slice with chocolate chip | Cake slice with chocolate chip | CHOCOLATE COATED CAKE BARS, INDIVIDUAL, PURCHASED | SRD has CHOCOLATE CAKE BAR WITH CHOCOLATE CHIPS PURCHASED, L0FoodCode==8161 and CHOCOLATE CHIP CAKES MADE WITH PUFA MARGARINE, HOMEMADE, L0FoodCode==860. | Changed FoodNumber to 8606 in SHeS and NDB |
| 5592 | Bobotie, lamb curry | Lamb bhuna, curry | LAMB CURRY ( NO POTATOES ) WITH ONIONS & CURRY PAS | SRD has "OBOTIE" (LAMB CURRY WITH EGGS AND MILK), L0FoodCode==6919.  SHeS doesn't have bobotie, but has a more general lamb curry 'Lamb curry, homemade 5592' | Assigned 'LAMB CURRY ( NO POTATOES ) WITH ONIONS & CURRY PAS' FoodNumber 6919 in SRD |
| 6919 | Lamb pasanda, curry | Lamb pasanda, curry | "BOBOTIE" (LAMB CURRY WITH EGGS AND MILK) | SRD has LAMB AND ONION CURRY, L0FoodCode==7120 and LAMB CURRY ( NO POTATOES ) WITH ONIONS & CURRY PAS, L0FoodCode==5592. | Assigned '"BOBOTIE" (LAMB CURRY WITH EGGS AND MILK)' FoodNumber 5592 in SRD |
| 5607 | Celery soup | Butternut squash soup | VEGETABLE SOUP NO POTATOES | SRD has SOUP WITH CELERY, BEANS & OLIVE OIL, L0FoodCode==2936 and CARROT AND CELERY SOUP, NO FAT, L0FoodCode==3268 and CELERY AND COTTAGE CHEESE SOUP, L0FoodCode==9192, and CELERY AND ONION SOUP, L0FoodCode==6504, and CELERY AND ONION SOUP WITH PUFA OIL, L0FoodCode==3628, and CELERY CHOWDER, L0FoodCode==9831, and CELERY, TOMATO, CARROT AND ONION SOUP, L0FoodCode==4088. | Assigned 'Butternut squash soup' FoodNumber 3955 in SHeS |
| 6736 | Butternut squash soup |  | PUMPKIN SOUP WITH ONION AND CREAM | SRD has BUTTERNUT SQUASH SOUP, L0FoodCode==3955. | Assigned 'Butternut squash soup' FoodNumber 3955 in NDB |
| 8136 | Kelloggs Special K Biscuit Moments | Kelloggs Special K Biscuit Moments | GO AHEAD YOGURT BREAKS | SRD has SPECIAL K CEREAL BARS, FRUIT WITH YOGURT TOPPING ONLY, L0FoodCode==10187. | Assigned 'Kelloggs Special K Biscuit Moments' FoodNumber 10187 in SHeS and NDB |
| 8445 | 7 Up free / light | 7 Up free / light | FRUIT JUICE DRINK <50% JUICE LOW CAL NOT CANNED | SRD has CARBONATED BEVERAGE NO JUICE CANNED LOW CALORIE', L0FoodCode==7902. | Assigned '7 Up free / light' FoodNumber 7902 in SHeS and NDB |
| 9372 | Challah bread | Continental bread (not ciabatta/focaccia/panini) | CONTINENTAL BREADS EG. CIABATTA FOCACCIA | Challah has egg so more like brioche in SRD, L0FoodCode==9129. | Assigned 'Challah bread' FoodNumber 9129 in NDB |

| **Supplemental Table S4.** Classification of milk dairy items. | | | | | |
| --- | --- | --- | --- | --- | --- |
| Food Number | Food Description | Milk | Skimmed Milk | Semi Skimmed Milk | Full Fat Milk |
| 601 | BUTTERMILK | 1 | 1 |  |  |
| 602 | MILK WHOLE SUMMER PASTEURISED | 1 |  |  | 1 |
| 603 | MILK WHOLE PASTEURISED WINTER | 1 |  |  | 1 |
| 604 | MILK WHOLE STERILISED | 1 |  |  | 1 |
| 605 | MILK WHOLE UHT | 1 |  |  | 1 |
| 608 | MILK SEMI-SKIMMED PASTEURISED SUMMER | 1 |  | 1 |  |
| 613 | MILK SKIMMED PASTEURISED SUMMER | 1 | 1 |  |  |
| 615 | MILK SKIMMED STERILIZED | 1 | 1 |  |  |
| 616 | MILK SKIMMED UHT | 1 | 1 |  |  |
| 622 | MILK EVAPORATED | 1 |  |  | 1 |
| 4713 | EVAPORATED MILK LOW FAT CANNED | 1 |  | 1 |  |
| 8149 | DRIED SKIMMED MILK POWDER | 1 | 1 |  |  |
| 8543 | MILK SEMI-SKIMMED PASTEURISED WINTER | 1 |  | 1 |  |
| 8544 | MILK SKIMMED PASTEURISED WINTER | 1 | 1 |  |  |
| 9132 | MILK SEMI SKIMMED STERILISED | 1 |  | 1 |  |
| 9218 | WHOLE MILK DRIED | 1 |  |  | 1 |
| 9493 | LACTOSE FREE SEMI SKIMMED MILK | 1 |  | 1 |  |
| 10251 | ONE PERCENT (1%) MILK, PASTEURISED | 1 |  | 1 |  |
| 10277 | ONE PERCENT (1%) MILK, PASTEURISED, HEATED/BOILED | 1 |  | 1 |  |
| 10397 | SEMI-SKIMMED GOATS MILK, PASTEURISED | 1 |  | 1 |  |
| 10498 | SEMI-SKIMMED DRIED MILK POWDER | 1 |  | 1 |  |
| 10932 | LACTOSE FREE WHOLE MILK | 1 |  |  | 1 |
| 11606 | LACTOSE FREE SKIMMED MILK | 1 | 1 |  |  |
| 7945 | SMA FOLLOW-ON FORMULA MILK, DRY WEIGHT (FORMERLY PROGRESS) | 1 |  |  | 1 |
| 10605 | SMA TODDLER MILK, 1 YEAR+, DRY WEIGHT ONLY | 1 |  |  | 1 |
| 10621 | APTAMIL GROWING-UP MILK FORMULA, TODDLERS 1 YEAR+, DRY WEIGHT | 1 |  |  | 1 |
| 10370 | COW AND GATE COMFORT MILK, DRY WEIGHT | 1 |  |  | 1 |
| 30084 | COW AND GATE GROWING UP MILK, 2-3 YEARS, DRY | 1 |  |  | 1 |

| **Supplemental Table S5.** Classification of cheese dairy items. | | | | | | | | |
| --- | --- | --- | --- | --- | --- | --- | --- | --- |
| Food Number | Food Description | Cheese | Cheddar Cheese | Cottage Cheese | Other Cheese | Skimmed Cheese | Semi Skimmed Cheese | Full Fat Cheese |
| 651 | CHEESE CAERPHILLY | 1 |  |  | 1 |  |  | 1 |
| 652 | CHEESE CAMEMBERT | 1 |  |  | 1 |  |  | 1 |
| 654 | CHEESE CHEDDAR ENGLISH | 1 | 1 |  |  |  |  | 1 |
| 659 | CHEESE CHEDDAR LOW FAT | 1 | 1 |  |  |  | 1 |  |
| 661 | CHEESE CHEDDAR ANY OTHER OR FOR RECIPES | 1 | 1 |  |  |  |  | 1 |
| 668 | CHEESE EDAM FULL FAT | 1 |  |  | 1 |  |  | 1 |
| 669 | CHEESE FETA | 1 |  |  | 1 |  |  | 1 |
| 670 | CHEESE GOUDA | 1 |  |  | 1 |  |  | 1 |
| 671 | CHEESE GRUYERE | 1 |  |  | 1 |  |  | 1 |
| 672 | CHEESE LEICESTERSHIRE | 1 |  |  | 1 |  |  | 1 |
| 675 | CHEESE MOZZARELLA | 1 |  |  | 1 |  |  | 1 |
| 676 | CHEESE PARMESAN | 1 |  |  | 1 |  |  | 1 |
| 679 | CHEESE STILTON BLUE | 1 |  |  | 1 |  |  | 1 |
| 683 | CHEESE WENSLEYDALE | 1 |  |  | 1 |  |  | 1 |
| 687 | COTTAGE CHEESE PLAIN | 1 |  | 1 |  |  |  | 1 |
| 688 | CHEESE CREAM FULLFAT | 1 |  |  | 1 |  |  | 1 |
| 691 | CHEESE BRIE | 1 |  |  | 1 |  |  | 1 |
| 2703 | CHEESE SOFT FULL FAT. PHILADELPHIA TYPE | 1 |  |  | 1 |  |  | 1 |
| 6958 | CHEESE PORT SALUT/ST PAULIN | 1 |  |  | 1 |  |  | 1 |
| 6978 | CHEESE MASCARPONE | 1 |  |  | 1 |  |  | 1 |
| 6979 | CHEESE SOFT LOW FAT | 1 |  |  | 1 |  | 1 |  |
| 6980 | CHEESE PANEER | 1 |  |  | 1 |  |  | 1 |
| 6981 | CHEESE HALLOUMI | 1 |  |  | 1 |  |  | 1 |
| 7057 | CHEESE GOATS FULL FAT | 1 |  |  | 1 |  |  | 1 |
| 7112 | CHEESE SOFT MEDIUM FAT | 1 |  |  | 1 |  | 1 |  |
| 7727 | CHEESE EDAM REDUCED FAT | 1 |  |  | 1 |  | 1 |  |
| 7728 | RICOTTA | 1 |  |  | 1 |  | 1 |  |
| 7734 | QUARK | 1 |  |  | 1 | 1 |  |  |
| 7735 | FROMAGE FRAIS, FULL FAT, NATURAL, UNSWEETENED | 1 |  |  | 1 |  |  | 1 |
| 7738 | FROMAGE FRAIS, VIRTUALLY FAT FREE, NATURAL, UNSWEET | 1 |  |  | 1 | 1 |  |  |
| 10543 | CHEDDAR REDUCED FAT 21-23G/100G | 1 | 1 |  |  |  | 1 |  |
| 10590 | LOW FAT MOZZARELLA | 1 |  |  | 1 |  | 1 |  |
| 10958 | LACTOSE FREE SOFT WHITE CHEESE | 1 |  |  | 1 |  |  | 1 |
| 10977 | FROMAGE FRAIS, LOW FAT, NATURAL, UNSWEETENED E.G., PETIT FILOUS | 1 |  |  | 1 |  | 1 |  |
| 11258 | COTTAGE CHEESE, NO FAT | 1 |  | 1 |  | 1 |  |  |

| **Supplemental Table S6.** Classification of yogurt, cream and butter dairy items. | | | | | | | | | |
| --- | --- | --- | --- | --- | --- | --- | --- | --- | --- |
| Food Number | Food Description | Yogurt | Skimmed Yogurt | Semi Skimmed Yogurt | Full Fat Yogurt | Cream | Semi Skimmed Cream | Full Fat Cream | Butter |
| 702 | YOGURT, WHOLE MILK, NATURAL, UNSWEETENED | 1 |  |  | 1 |  |  |  |  |
| 705 | YOGURT LOW FAT NATURAL UNSWEETENED | 1 |  | 1 |  |  |  |  |  |
| 710 | YOGURT GOAT MILK | 1 |  |  | 1 |  |  |  |  |
| 2700 | YOGURT, VIRTUALLY FAT FREE, NATURAL, UNSWEETENED | 1 | 1 |  |  |  |  |  |  |
| 7741 | YOGURT, GREEK STYLE, COWS, NATURAL, WHOLE MILK | 1 |  |  | 1 |  |  |  |  |
| 7742 | GREEK YOGURT SHEEP | 1 |  |  | 1 |  |  |  |  |
| 11249 | YOGURT VIRTUALLY FAT FREE, NATURAL, STRAINED (HIGH PROTEIN) | 1 | 1 |  |  |  |  |  |  |
| 632 | CREAM CLOTTED |  |  |  |  | 1 |  | 1 |  |
| 633 | CREAM SOURED |  |  |  |  | 1 |  | 1 |  |
| 634 | CREAM DOUBLE |  |  |  |  | 1 |  | 1 |  |
| 636 | CREAM HALF PASTEURISED |  |  |  |  | 1 | 1 |  |  |
| 638 | CREAM SINGLE PASTEURISED |  |  |  |  | 1 | 1 |  |  |
| 639 | CREAM SINGLE FROZEN |  |  |  |  | 1 | 1 |  |  |
| 640 | CREAM SINGLE UHT |  |  |  |  | 1 | 1 |  |  |
| 643 | CREAM DAIRY UHT AEROSOL |  |  |  |  | 1 |  | 1 |  |
| 644 | CREAM WHIPPING FRESH |  |  |  |  | 1 |  | 1 |  |
| 645 | CREAM WHIPPING FROZEN |  |  |  |  | 1 |  | 1 |  |
| 646 | CREAM WHIPPING UHT |  |  |  |  | 1 |  | 1 |  |
| 2681 | ELMLEA IMITATION DOUBLE CREAM |  |  |  |  | 1 | 1 |  |  |
| 3014 | CREME FRAICHE |  |  |  |  | 1 |  | 1 |  |
| 4328 | EMLEA IMITATION CREAM SINGLE |  |  |  |  | 1 | 1 |  |  |
| 6984 | CREAM, DAIRY, EXTRA THICK, 24% FAT |  |  |  |  | 1 |  | 1 |  |
| 6985 | CREME FRAICHE, HALF FAT (15%) |  |  |  |  | 1 | 1 |  |  |
| 6986 | CREAM, DAIRY, UHT, AEROSOL, HALF FAT |  |  |  |  | 1 | 1 |  |  |
| 852 | BUTTER UNSALTED |  |  |  |  |  |  |  | 1 |
| 856 | GHEE MADE FROM BUTTER |  |  |  |  |  |  |  | 1 |

| **Supplemental Table S7.** Percent contribution of food groups to dairy intake among adult dairy consumers (16+ years) in the Scottish Health Survey, 2021 (unweighted sample size: 3,425). | | |
| --- | --- | --- |
|  | **Mean** | **SD** |
| SEMI SKIMMED MILK | 28.5 | 34.5 |
| OTHER MILK AND CREAM | 10.4 | 24.1 |
| WHOLE MILK | 9.1 | 23.8 |
| YOGURT FROMAGE FRAIS AND DAIRY DESSERTS | 8.7 | 19.1 |
| CHEESE | 6.7 | 15.3 |
| PASTA RICE AND OTHER CEREALS | 5.4 | 16.5 |
| SKIMMED MILK | 5 | 16.5 |
| SANDWICHES | 3.5 | 12.1 |
| BUTTER | 2.9 | 8.6 |
| HIGH FIBRE BREAKFAST CEREALS | 2 | 9.6 |
| EGGS AND EGG DISHES | 1.8 | 8.9 |
| ICE CREAM | 1.6 | 7.4 |
| PUDDINGS | 1.4 | 7.2 |
| MISCELLANEOUS | 1.4 | 7.1 |
| CHOCOLATE CONFECTIONERY | 1.3 | 6.3 |
| 1% Fat Milk | 1.3 | 8.8 |
| BISCUITS | 1.1 | 5.6 |
| BUNS CAKES PASTRIES & FRUIT PIES | 1.1 | 4.6 |
| CHICKEN AND TURKEY DISHES | 1 | 5.7 |
| VEGETABLES NOT RAW | 1 | 7.4 |
| WHITE BREAD | 0.9 | 5.8 |
| BEEF VEAL AND DISHES | 0.8 | 5.3 |
| OTHER WHITE FISH SHELLFISH & FISH DISHES | 0.6 | 5 |
| REDUCED FAT SPREAD | 0.5 | 4.6 |
| OTHER POTATOES POTATO SALADS & DISHES | 0.4 | 3.1 |
| MEAT PIES AND PASTRIES | 0.3 | 3.1 |
| BURGERS AND KEBABS | 0.2 | 2.1 |
| WHOLEMEAL BREAD | 0.1 | 1.2 |
| LOW FAT SPREAD | 0.1 | 1.9 |
| LAMB AND DISHES | 0.1 | 1.7 |
| COATED CHICKEN | 0.1 | 1 |
| WHITE FISH COATED OR FRIED | 0.1 | 2.1 |
| CHIPS FRIED & ROAST POTATOES AND POTATO PRODUCTS | 0.1 | 3.2 |
| SUGARS PRESERVES AND SWEET SPREADS | 0.1 | 2.4 |
